# Supplementary material for: Isolation, engineering and ecology of temperate phages from the human gut
Source: Nature. 2025 Oct 15;647(8090):698–705. doi: 10.1038/s41586-025-09614-7 (PMC12629997; doi:10.1038/s41586-025-09614-7)
Supplement: Supplementary file 1 — The legends for Supplementary Tables 1–12. [file 41586_2025_9614_MOESM1_ESM.pdf]

nature portfolio

https://doi.org/10.1038/s41586-025-09614-7

Supplementary information

Isolation, engineering and ecology of temperate phages from the human gut

In the format provided by the authors and unedited

Supplementary Tables

Supplementary Table 1. Bacterial isolate information.

AusMiCC identifier, ENA accession number, prophage and taxonomic information of isolates used in this study.

Supplementary Table 2. Induction sample sequencing information.

AusMiCC identifier and sample ID for all single isolate induction samples.

Supplementary Table 3. Inducible prophage genomes summary.

ENA genome assembly and read ERR identifier, species group and host taxonomic information of induced prophages. Presence of DGR variable repeats and viral hallmark genes as well as vContact2 viral cluster.

Supplementary Table 4. vContact2 cluster information.

vContact2 clusters and annotations of induced prophages plus phage database genomes

Supplementary Table 5. Gut virome metadata.

Metadata of gut viromes used in this study to determine prevalence of induced prophages and database phage genomes.

Supplementary Table 6. Prophage lengths and PHROG annotations.

Induced and predicted prophage gene annotations.

Supplementary Table 7. Prophage high similarity pairs and whole genome dN/dS.

Whole genome dN/dS data of induced and predicted prophages. High similarity pairs annotated and HGT and insertion/deletion events shown for these pairs.

Supplementary Table 8. Host dN/dS.

Whole genome host dN/dS comparisons.

Supplementary Table 9 Prophage genes dN/dS.

Per gene dN/dS of induced and predicted prophages. High similarity phage pairs annotated.

Supplementary Table 10. Plasmids and primers.

Plasmids and primers used in this study. Sanger sequencing result of CC01414 Δtran.

Supplementary Table 11. Induction qPCR.

Phage qPCR data of phage Pomma in CC01414 and CC01414 Δtran, as well as phage Pomma and Wilby in CC01407, CC01390, CC1401, CC01404 and CC01405.

Supplementary Table 12. ENA accession information.

ENA accession number of all samples sequenced in this study.
