# Supplementary material for: Isolation, engineering and ecology of temperate phages from the human gut
Source: Nature. 2025 Oct 15;647(8090):698–705. doi: 10.1038/s41586-025-09614-7 (PMC12629997; doi:10.1038/s41586-025-09614-7)
Supplement: Supplementary file 2 — Reporting Summary [file 41586_2025_9614_MOESM2_ESM.pdf]

nature portfolio

Corresponding author(s): Jeremy J. Barr

Last updated by author(s): Aug 20, 2025

Reporting Summary

Nature Portfolio wishes to improve the reproducibility of the work that we publish. This form provides structure for consistency and transparency in reporting. For further information on Nature Portfolio policies, see our Editorial Policies and the Editorial Policy Checklist.

Statistics

For all statistical analyses, confirm that the following items are present in the figure legend, table legend, main text, or Methods section.

n/a	Confirmed	
☐	☒	The exact sample size (*n*) for each experimental group/condition, given as a discrete number and unit of measurement
☐	☒	A statement on whether measurements were taken from distinct samples or whether the same sample was measured repeatedly
☐	☒	The statistical test(s) used AND whether they are one- or two-sided<br>*Only common tests should be described solely by name; describe more complex techniques in the Methods section.*
☒	☐	A description of all covariates tested
☐	☒	A description of any assumptions or corrections, such as tests of normality and adjustment for multiple comparisons
☐	☒	A full description of the statistical parameters including central tendency (e.g. means) or other basic estimates (e.g. regression coefficient) AND variation (e.g. standard deviation) or associated estimates of uncertainty (e.g. confidence intervals)
☐	☒	For null hypothesis testing, the test statistic (e.g. *F*, *t*, *r*) with confidence intervals, effect sizes, degrees of freedom and *P* value noted<br>*Give P values as exact values whenever suitable.*
☒	☐	For Bayesian analysis, information on the choice of priors and Markov chain Monte Carlo settings
☒	☐	For hierarchical and complex designs, identification of the appropriate level for tests and full reporting of outcomes
☐	☒	Estimates of effect sizes (e.g. Cohen's *d*, Pearson's *r*), indicating how they were calculated

Our web collection on statistics for biologists contains articles on many of the points above.

Software and code

Policy information about availability of computer code

Data collection	Presence of phage sized DNA in agarose gels were visualized using Image Studio Lite LI-COR Biosciences V5.2. qPCR data was collected using Roche Lightcycler® 480 system II release 1.5.1.62.
Data analysis	Software used for data analysis: SeqKit (v.2), MAFFT (v7.310), trimAI (v1.4.1), RAxML (v8.2.12), iTOL, dRep (v.3.0.0), Virsorter, Vibrant, VirFinder, CheckV, R IRanges (v.2.28.0), Trimmomatic (v.0.38), Bowtie2 (v.2.3.5), Samtools (v.1.9), Deeptools (v.3.1.3), hafeZ (v1.0.2), MetaViral SPade, PROKKA (v.1.14.6), Hmmer (v.3.3.1), TABAJARA, vContact2, DGRscan, BLAST (v.2.7.1+), splicejam (v 0.0.77), fastANI (v.1.33), Primer 3, dragonfly (v.1.0.14), unicycler (v. 0.4.7), RagTag, R rstatix (v.0.7.0), R ggpubr (v.0.4.0), R gggenomes (v.0.9.9.9000), ggplot and R base stats (R version 4.1.3), bbmap (v39.06), bedtools (v.26.0), KrakenUnique (v.1.0.4), Jellyfish (v1.1.12), RapidNJ (v2.3.2), Expam (v1.2.2.5)

For manuscripts utilizing custom algorithms or software that are central to the research but not yet described in published literature, software must be made available to editors and reviewers. We strongly encourage code deposition in a community repository (e.g. GitHub). See the Nature Portfolio guidelines for submitting code & software for further information.

Data

Policy information about availability of data

All manuscripts must include a data availability statement. This statement should provide the following information, where applicable:

- Accession codes, unique identifiers, or web links for publicly available datasets
- A description of any restrictions on data availability
- For clinical datasets or third party data, please ensure that the statement adheres to our policy

All data for this study have been deposited in the European Nucleotide Archive (ENA) at EMBL-EBI under project number PRJEB64565 and accession numbers are provided in Supplementary Table S1, S3 and S12. Bacterial isolates are available through the Australian Microbiome Culture Collection (AusMiCC). Metadata of viromes used in this study along with accession numbers are provided in Supplementary Table S5. Bioinformatic scripts and Figure data is available in Figshare DOI https://doi.org/10.26180/29946902.v1

Research involving human participants, their data, or biological material

Policy information about studies with human participants or human data. See also policy information about sex, gender (identity/presentation), and sexual orientation and race, ethnicity and racism.

Reporting on sex and gender	n/a
Reporting on race, ethnicity, or other socially relevant groupings	n/a
Population characteristics	n/a
Recruitment	n/a
Ethics oversight	n/a

Note that full information on the approval of the study protocol must also be provided in the manuscript.

Field-specific reporting

Please select the one below that is the best fit for your research. If you are not sure, read the appropriate sections before making your selection.

☒ Life sciences ☐ Behavioural & social sciences ☐ Ecological, evolutionary & environmental sciences

For a reference copy of the document with all sections, see nature.com/documents/nr-reporting-summary-flat.pdf

Life sciences study design

All studies must disclose on these points even when the disclosure is negative.

Sample size	No sample size calculation was performed due to exploratory nature of project.
Data exclusions	Low quality reads from Illumina sequencing were excluded using Trimmomatic (SLIDINGWINDOW:4:25 MINLEN:100). Possible human, phiX-174 and cloning vector contaminants were removed from metagenomes before read mapping to temperate phage genomes.
Replication	Sequencing of prophage induction in pure isolate samples were performed in singlet due to cost of high throughput screens. Sequencing of synthetic microbiome was performed on 5 biological replicates. Sequencing of gene deletion mutant strain (plus wilde-type) was performed in singlet and was validated by qPCR.<br>qPCR was performed in biological triplicates with technical triplicates for each sample, no failed replications occurred.
Randomization	Randomization is not relevant to the microbiological experiments performed in this study.
Blinding	Blinding during data collection is not relevant to the microbiological experiments performed in this study.

Reporting for specific materials, systems and methods

We require information from authors about some types of materials, experimental systems and methods used in many studies. Here, indicate whether each material, system or method listed is relevant to your study. If you are not sure if a list item applies to your research, read the appropriate section before selecting a response.

Materials & experimental systems

n/a	Involved in the study
☒	☐ Antibodies
☐	☒ Eukaryotic cell lines
☒	☐ Palaeontology and archaeology
☒	☐ Animals and other organisms
☒	☐ Clinical data
☒	☐ Dual use research of concern
☒	☐ Plants

Methods

n/a	Involved in the study
☒	☐ ChIP-seq
☒	☐ Flow cytometry
☒	☐ MRI-based neuroimaging

Eukaryotic cell lines

Policy information about cell lines and Sex and Gender in Research

Cell line source(s)	Human colonic epithelial immortalised cells (Caco2 TC7)
Authentication	Genotype verified by AGRF Human Cell Line Identification Service; VIC, Australia
Mycoplasma contamination	Cells were routinely tested for the presence of mycoplasma contamination (MycoStrip, Invivogen, San Diego, USA) and were confirmed mycoplasma negative.
Commonly misidentified lines (See ICLAC register)	No commonly misidentified cell lines listed in ICLAC register was used in this study.

Plants

Seed stocks	n/a
Novel plant genotypes	n/a
Authentication	n/a
